# Pilot Testing in the Wild: Feasibility, Acceptability, Usage Patterns, and Efficacy of an Integrated Web and Smartphone Platform for Bipolar II Disorder

**DOI:** 10.2196/32740

**Published:** 2022-05-31

**Authors:** Kathryn Fletcher, Katrina Lindblom, Elizabeth Seabrook, Fiona Foley, Greg Murray

**Affiliations:** 1 Centre for Mental Health Swinburne University of Technology Melbourne Australia

**Keywords:** bipolar disorder, smartphone, app, web-based intervention, ecological momentary assessment, mobile phone

## Abstract

**Background:**

Bipolar II disorder (BD-II) is associated with significant burden, disability, and mortality; however, there continues to be a dearth of evidence-based psychological interventions for this condition. Technology-mediated interventions incorporating self-management have untapped potential to help meet this need as an adjunct to usual clinical care.

**Objective:**

The objective of this pilot study is to assess the feasibility, acceptability, and clinical utility of a novel intervention for BD-II (Tailored Recovery-oriented Intervention for Bipolar II Experiences; TRIBE), in which mindfulness-based psychological content is delivered via an integrated web and smartphone platform. The focus of the study is evaluation of the dynamic use patterns emerging from ecological momentary assessment and intervention to assist the real-world application of mindfulness skills learned from web-delivered modules.

**Methods:**

An open trial design using pretest and posttest assessments with nested qualitative evaluation was used. Individuals (aged 18-65 years) with a diagnosis of BD-II were recruited worldwide and invited to use a prototype of the TRIBE intervention over a 3-week period. Data were collected via web-based questionnaires and phone interviews at baseline and 3-week follow-up.

**Results:**

A total of 25 participants completed baseline and follow-up assessments. Adherence rates (daily app use) were 65.6% across the 3-week study, with up to 88% (22/25) of participants using the app synergistically alongside the web-based program. Despite technical challenges with the prototype intervention (from user, hardware, and software standpoints), acceptability was adequate, and most participants rated the intervention positively in terms of concept (companion app with website: 19/25, 76%), content (19/25, 76%), and credibility and utility in supporting their management of bipolar disorder (17/25, 68%). Evaluation using behavioral archetypes identified important use pathways and a provisional model to inform platform refinement. As hypothesized, depression scores significantly decreased after the intervention (Montgomery-Asberg Depression Rating Scale baseline mean 8.60, SD 6.86, vs follow-up mean 6.16, SD 5.11; t_24_=2.63; P=.01; Cohen *d*=0.53, 95% CI 0.52-4.36).

**Conclusions:**

Our findings suggest that TRIBE is feasible and represents an appropriate and acceptable self-management program for patients with BD-II. Preliminary efficacy results are promising and support full development of TRIBE informed by the present behavioral archetype analysis. Modifications suggested by the pilot study include increasing the duration of the intervention and increasing technical support.

## Introduction

### Background

Bipolar disorder (BD) is a chronic mood disorder characterized by episodes of depression, hypomania or mania, and mixed mood states. Although historically perceived as the *milder* phenotype, bipolar II disorder (BD-II) is associated with comparable depression severity, suicide attempts, and role impairment [1] relative to bipolar I disorder (BD-I). Notably, poorer health-related quality of life [2] and a higher risk of completed suicides [3] have been observed in patients with BD-II. These concerning features may be because of the significant depressive burden characterizing the condition, including persistent subsyndromal depression, poorer return to baseline psychosocial functioning between episodes [4-6], and mixed depressive states that are experienced as highly dysphoric [7]. Despite the best available treatments, depression relapse rates remain high [8,9] and are more frequent in patients with BD-II [10]. Therefore, there is a pressing need for improved treatments.

Several adjunctive psychological interventions have been developed for BD, but research has overwhelmingly focused on BD-I, leaving clinicians to simply extrapolate the findings to BD-II. These treatments lack face validity because of their focus on the prevention of mania and relative inattention to elevated mood states of BD-II, which, by definition, do not meet the criteria for mania. Furthermore, in the absence of research into BD-II, clinical guidelines are reduced to tentative recommendations to generalize from BD-I [11], ignoring genetic evidence of their separability [12] and clear phenomenological differences among the phenotypes [1]. Beyond the core diagnostic difference (absence of mania), people with BD-II experience higher levels of residual depressive symptoms [13-15], more frequent depressive episodes [4,6,16], and shorter *well* periods [5,17]. Indeed, a highly cited prospective study found that people with BD-II experience depression 53% of the time (approximately 32% in BD-I) [18]. Existing evidence-based psychological interventions for BD generally have the greatest impact on depressive symptoms [19]; indeed, current National Institute for Health and Care Excellence treatment guidelines for people in the depressive phase of BD recommend offering evidence-based psychological treatments for unipolar depression [20]. The importance of psychological intervention for targeting depressive symptoms of BD is particularly germane to BD-II, given its predominant and impairing syndromal and subsyndromal depression. There is growing recognition that the salience of psychotherapies for this underserved group may improve if modified specifically for BD-II [21-26] and that self-management can complement therapist-delivered interventions for BD [27-29]. Self-management is increasingly seen as a cornerstone of effective treatment, empowering individuals to play an active role in managing their condition and improving their quality of life [30,31]. Digital mental health platforms can improve access to these empowering activities [28].

### The Role of Technology: Computers Versus Smartphones

The web provides economical access to tailored psychological interventions, and specifically in BD, it can overcome many barriers to accessing psychological assistance, including cost, time, and trust in professionals [31]. Web-based therapies are acceptable to patients with BD [32]. People with BD-II are already proactively seeking information on the web, and the web has untapped potential to provide worldwide access to evidence-based treatment for BD-II. Web-based interventions in mental health have primarily been investigated through websites accessed through computers.

Few studies have focused on the clinical utility of smartphones in people with serious mental illnesses. There is evidence that smartphone technology is perceived by patients as an acceptable, time-unconstrained, user-friendly, and noninvasive tool for mental health self-management [33]. In an investigation of those, aged between 18 and 30 years, with BD, 79% of those not using smartphone apps to manage their condition were interested in trying them, whereas 61% of the self-management strategies provided by these apps were viewed as desirable by the study participants [34].

Smartphones would seem to have particular application in targeting the dynamic, chronic BD-II illness course. Studies using daily smartphone-based self-monitoring have observed more fluctuations and depressive symptoms in BD-II than in BD-I [35]. Smartphone apps can facilitate regular, unobtrusive mood monitoring (through push notifications) to increase awareness of subsyndromal mood fluctuations that may herald an oncoming mood episode but can be extended further to act as a *therapist-in-the-pocket* via delivery of mood-specific coping strategies promoting in-the-moment intervention that is tailored to the individual. The clinical utility of this ecological momentary assessment and intervention (EMA/I) approach has been demonstrated in the treatment of psychotic disorders [36] and as an add-on treatment for those with mild to severe unipolar depression including residual depressive states [37].

A growing number of studies have adopted EMA/I in BD. Hidalgo-Mazzei et al [38] evaluated mood monitoring with the provision of personalized psychoeducation-based prompts via a smartphone app (SIMPLe), demonstrating feasibility and acceptability in patients with BD as an adjunct to usual clinical care [38]. A similar app developed for BD (PRISM), augmenting brief (face-to-face) psychoeducation treatment, demonstrated feasibility, high levels of acceptability and satisfaction, and high retention rates (93% at 12 weeks) in a randomized single-blind controlled trial with 82 participants with BD (10/82, 12% BD-II). A significant effect on depressive symptom severity was found, favoring PRISM over active control [39]. Although promising, critical consideration and further clarification of the role of EMA/I in BD are required, with negative study findings in relation to reduction of depressive symptoms reported in one study (MONARCA I) [40]. Two key limitations of research to date are notable. First, studies have been weighted to BD-I samples. Therefore, the utility of EMA/I for BD-II requires investigation. Second, EMA/I prompts primarily focus on psychoeducational content within a symptom-focused relapse prevention framework. We previously argued that such frameworks may be demoralizing for some individuals with BD, particularly those who have experienced multiple episodes [32]. A recovery-oriented approach recognizing the unavoidability of suffering, emphasizing the redefinition of life goals, and prioritizing other outcomes (eg, quality of life and functioning) may be more beneficial for BD [41,42]. These priorities are consistent with mindfulness-based interventions (MBIs), which elevate mindful awareness of present experience and a nonjudgmental stance toward that experience. As reviewed previously, MBIs hold particular promise for BD [43], with preliminary evidence supporting their benefits for depression, anxiety, and mood regulation in BD [44]. Mindfulness-based EMA/I approaches have not been tested for BD and have a strong potential utility for BD-II.

### This Study

This study aimed to evaluate a mindfulness-based digital intervention for BD-II, delivered via a novel integrated web and smartphone platform. The objective was to assess the feasibility, acceptability, and clinical utility of the intervention. Building on our prior research into web-based psychological interventions for BD [32,41,45], a low-intensity, web-based self-help program with a companion smartphone app was developed to improve depression and related outcomes in those with BD-II: the Targeted Recovery-oriented Intervention for Bipolar II Experiences (TRIBE). We hypothesized that the intervention would reduce depression, hypomania, and anxiety severity and improve quality of life, emotion regulation, and mindfulness. The rationale for the hybrid intervention emerged from device-sensitive thinking in digital health. We postulated that an optimal digital platform for the marked symptom variation in BD-II would use the complementary strengths of computer or websites and smartphone or app devices. Computer devices are preferred for more extensive engagement with information (viewing videos, writing, undertaking reflective exercises as in traditional eTherapies), whereas apps can prompt monitoring and new behaviors in everyday life [46].

Evaluation was conducted through an open pilot trial of a prototype version of the intervention and took 3 forms. First, feasibility and acceptability were assessed using quantitative and qualitative self-reports. Second, individual differences in engagement with various features and pathways of the intervention were explored using an analysis of behavioral archetypes [47]. Finally, a preliminary investigation of clinical utility was conducted by exploring intervention-related changes in clinician-rated depression scores (primary outcome) and hypomania, anxiety, mindfulness, and emotion regulation (secondary outcomes).

## Methods

### Study Design

The intervention was tested using a pretest-posttest open trial design with a nested qualitative evaluation. Feasibility and acceptability were assessed via (1) retention to the study (including baseline, intervention, and follow-up) and reasons for ineligibility or withdrawal, (2) level of adherence to the intervention, and (3) detailed participant feedback exploring the experience of and satisfaction with the intervention. To justify and guide the design of a later larger-scale evaluation, the most appropriate primary outcome measures were explored, informed by preliminary efficacy data and effect size estimates.

### Ethics Approval

This study was approved by the Swinburne Human Research Ethics Committee (SHR 2018/324).

### Procedure

To demonstrate international scalability, participants were recruited worldwide via three sources: (1) research participants from bipolar-specific studies (with international participants) conducted by Swinburne University of Technology who agreed to be contacted for future research, (2) contacts associated with a local community group (Bipolar Life), and (3) social media (Facebook and Twitter).

Inclusion and exclusion criteria were minimally restrictive to maximize generalizability of results. Inclusion criteria were as follows: (1) aged 18 to 65 years; (2) diagnosed with BD-II by a medical practitioner; (3) confirmation of the Diagnostic and Statistical Manual of Mental Disorders, Fourth Edition diagnosis of BD-II as assessed by telephone interview with the Mini International Neuropsychiatric Interview (MINI) [48]; (4) ready daily access to the internet and a smartphone and adequate literacy with both technologies; (5) sufficient understanding of written and spoken English to provide consent, engage with interviews, and use the intervention; and (6) under the care of a nominated medical practitioner (at least one contact within the past 12 months) and able to provide contact details. Exclusion criteria were as follows: (1) Diagnostic and Statistical Manual of Mental Disorders, Fourth Edition diagnosis of BD-I, schizoaffective disorder, or schizophrenia (assessed by MINI); (2) experiencing a current acute episode of depression or hypomania or being in a current psychotic episode (assessed by MINI); and (3) experiencing current acute suicidality, assessed using the Columbia Suicide Severity Rating Scale [49].

Individuals interested in participating were invited to the study website to register their details (including those of their medical health professional) and provide informed consent. The participants were then contacted by the research team to complete baseline phone interviews to determine eligibility. Eligible participants then completed a battery of web-based self-report measures and were provided with instructions on how to use the app via email and an access code for the study website. Following the 3-week intervention, participants were invited to complete the postintervention assessment, including a phone interview, self-report measures, and an optional 20- to 30-minute qualitative feedback phone interview. Participants were reimbursed for each assessment (US $25) and an additional US $25 for participation in the qualitative feedback interview.

Risk management procedures were followed according to a protocol developed as part of a prior randomized controlled trial testing the effectiveness of a web-based psychological intervention for BD [32]. As an adjunct to usual clinical care, participants were advised that the intervention would not provide emergency support (ie, website and app not monitored in real time) and as part of the inclusion criteria, were required to consent to the research team contacting their medical practitioner as required.

### Intervention

#### Overview

The overarching rationale for TRIBE’s integration of the 2 technologies was that a well-designed hybrid intervention might generate synergistic benefits for BD-II. Specifically, it was conjectured that smartphone-mediated intervention could help people moderate symptoms of depression and hypomania (real-time intervention *augments* coping) and support real-world generalization of skills, extending the reach of the web-delivered therapeutic content (real-time intervention *supports development* of new coping skills) through an EMA/I approach. We postulated that the combination of learning (so-called *offline cognition* [50], optimally delivered through a structured program of web-based materials) and doing (*online cognition*, optimally delivered through a smartphone app) and crosstalk between the 2 technologies would create a novel digital ecosystem with benefits for self-management of BD.

Next we describe the intervention’s 2 technologies separately (web-based program and smartphone app) before describing their integration in the hybrid platform.

#### Web-Based Program

**Figure 1:**
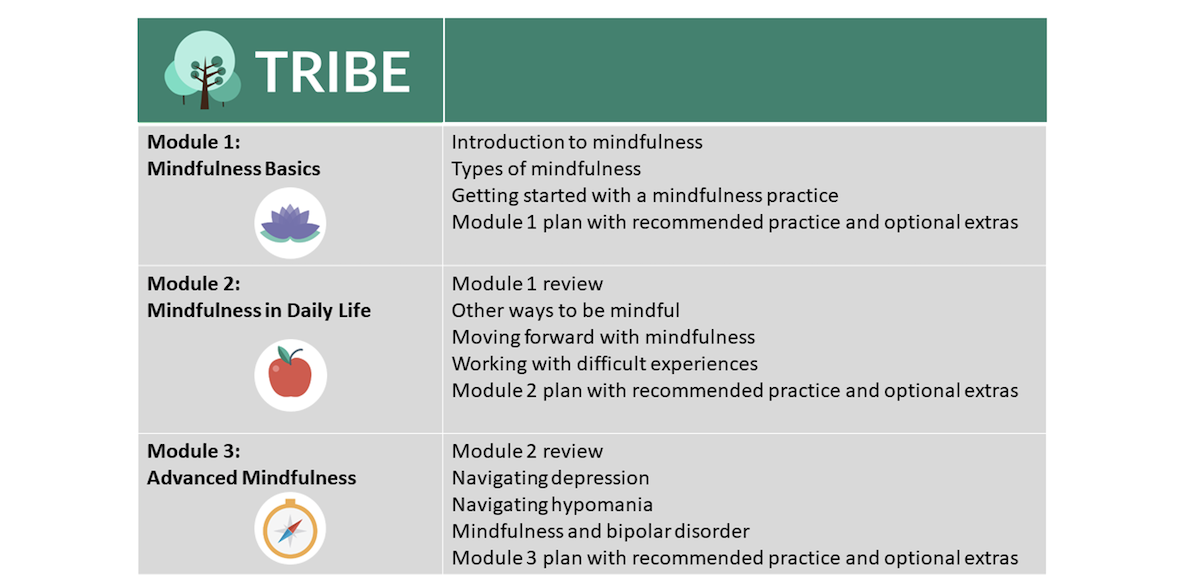
Content of the web-based program. TRIBE: Tailored Recovery-oriented Intervention for Bipolar II Experiences.

TRIBE includes a 3-week web-based intervention fostering the development of mindfulness skills, adapted from an earlier standalone web intervention designed to improve quality of life in those with any of the BDs [32,41]. The program is entirely self-guided, wherein participants complete modules at their own pace and can return to previous modules over the course of 3 weeks. The program consists of 3 modules, as illustrated in Figure 1.

In the iteration tested here, participants were encouraged to complete one module per week over the 3-week intervention period. To help build mindfulness skills, daily practice was recommended with a suggested homework exercise provided at the end of each module. A range of optional mindfulness exercises was offered to cater to participants with differing levels of mindfulness experience. Detailed descriptions of persuasive system design features and content based on a related intervention can be found in the studies by Fletcher et al [32,45].

#### Smartphone App

Owing to time and financial constraints, a pre-existing smartphone app was configured for use in this pilot test of TRIBE. MoodPrism (available for iOS and Android) was designed for mood tracking and monitoring emotional states in real time within everyday contexts [51,52]. MoodPrism is derived from research into EMA/I [53]. Here, MoodPrism also functioned to direct participants to, and remind them of, the programmatic content they were concurrently being offered through the web-based program.

### Rationale and Design of an Integrated Website-App Ecosystem

The digital design translation of this rationale was as follows. To encourage real-world application of mindfulness skills learned from the web-based program, the MoodPrism app was configured to send twice-daily notifications (at random times, during a specified minimum 12-hour time window selected by the user) to prompt a mood *check-in* to capture real-time assessments of emotional well-being, event-related experiences, and user context. Mood check-ins comprised 4 items on depression and anxiety (drawn from the 4-item Patient Health Questionnaire), 3 items on emotional state (positive and negative affect, arousal, and control) based on the Circumplex Model of Emotion [54], and 5 items on positive functioning (social connection, motivation, life meaning, self-esteem, and sense of achievement). Users were then asked if a notable event had occurred since the last check-in (with example event categories provided [51]) and to rate the event’s affective strength (from unpleasant to pleasant). The context (social, environmental, and behavioral) was assessed using single items. Following a mood check-in, participants were provided with a description of their current mood state (eg, *your mood check-in suggests you are feeling excited*) and in what context.

Critically, for integration of the 2 technologies, the MoodPrism app included the capability for participants to click on a hyperlink that redirected them to the web-based program (this step required log-in to the website) to receive a specific coping suggestion (Table 1 provides coping suggestion examples) based on their current mood state. Two types of coping suggestions were provided for each mood state, *Try it now* suggestions, which were actions to do in the moment, and *Learn more* suggestions, which were a way of re-engaging users with web-based content (eg, redirection to a mindfulness exercise learned within the context of a specific module), to consolidate knowledge and application in daily life.

**Figure 2:**
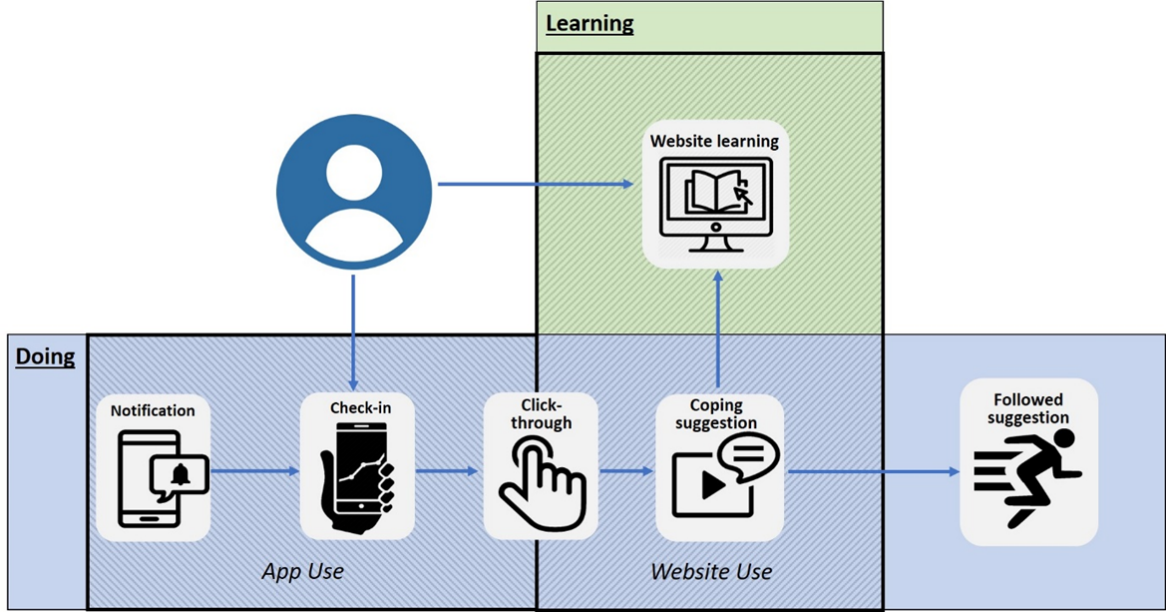
Tailored Recovery-oriented Intervention for Bipolar II Experiences ideal user flow journey.

The anticipated ideal user interactive path model [55], ideal user flow, and ideal user journey map for the use of TRIBE elements is shown in Figure 2.

**Table 1:** Coping suggestion examples.

Mood state	Coping suggestion
Exhausted or drained	“Go for a brief walk (up to 10 minutes or longer if you wish). As you are walking, consciously notice and acknowledge as many pleasant things as possible: smiling strangers, birds chirping, the feeling of the sun on your skin (or interesting cloud formations), a cat walking across the stress, flowers, etc. Focus on those as much as possible. Notice how you feel after your walk.”
Anxious or on edge	“Find a relaxing song, plug in some headphones, and take a quick listening break. Pay attention to each of the sounds you hear in the song. When you notice thoughts come into your mind, bring yourself back to sounds in the song (instruments, voice, beat). Music has powerful emotional effects—relaxing music can help you feel calmer.”
In balance	“Do a fake yawn (this will trigger a real yawn). Say ‘ahh’ as you exhale. Notice how a yawn interrupts thoughts and feelings and brings you into the present moment. Then, stretch very slowly for at least 10 seconds. Notice any tightness in your body. Bring your arms down by your sides and roll your shoulders backwards slowly, then forwards, noticing what this feels like. End with another yawn. Take a few moments to notice how you feel.”
Excited or energized	“If you’re feeling a bit excited or wired, it might be time to bring things back into balance. Try this simple breathing exercise to slow things down. Take 10 slow breaths—on each in-breath pause and hold for a few seconds, then release. Notice your belly rise and fall as you breathe. Count each breath up to 10. If you lose count, start again.”

### Measures

Clinician- and self-reported measures (conducted over the telephone and on the web, respectively) were administered at baseline and at the 3-week follow-up (immediately after the intervention).

#### Clinician-Rated Depression

The Montgomery-Asberg Depression Rating Scale (MADRS) [56] is a widely used, valid, and reliable 10-item clinician-administered scale for depression symptoms that is sensitive to change. Higher scores indicate more severe depression. Although the MADRS contains an item (*apparent sadness*) based on visual observation, prior studies have demonstrated that the full MADRS, including this item, can be administered reliably via telephone [57,58].

#### Self-rated Depression

The 16-item Quick Inventory of Depressive Symptomatology-Self-Report [59] is a self-report measure of depression symptoms with high internal consistency (Cronbach α=.87) that correlates strongly with established clinician-rated scales including the Hamilton Depression Rating Scale (*r*=0.86) [59]. Higher scores indicate more severe depression.

#### Hypomania

The Young Mania Rating Scale (YMRS) [60] is an 11-item clinician-administered scale that assesses the symptoms of hypomania and mania. This scale has well-established reliability and validity [60,61]. Higher scores indicate more severe mania. The Altman Self-Rating Mania Scale [62] is a 5-item self-reported measure of hypomanic and manic symptoms. Individuals are asked to choose a statement out of 5 for each item that best describes how they have been feeling over the past week. Scores >5 indicate hypomania or mania, with higher scores representing higher severity. The measure has shown good test-retest reliability (*r*=0.86-0.89) and appropriate concurrent validity, supported by correlations with interviewer-rated scales (eg, YMRS) [62].

#### Anxiety

The Depression Anxiety Stress Scale (DASS)-21 items [63] is a valid and reliable self-report measure of symptoms experienced over the previous week. The DASS-Anxiety subscale score was used in this study, measuring anxiety symptoms including physiological arousal and fear-based emotions. High internal consistency (Cronbach α=.87) has been reported for this subscale [64]. Higher scores indicate more severe anxiety.

#### Quality of Life

Subjective quality of life was measured using the self-report Brief Quality of Life in Bipolar Disorder (QoL.BD) scale [65]. Satisfaction with functioning is rated on a 5-point Likert scale, with higher scores representing greater satisfaction. The QoL.BD has adequate internal reliability (Cronbach α=.87) and appropriate test-retest reliability and is comparable with similar measures [65].

#### Emotion Regulation

The self-reported Difficulties in Emotion Regulation Scale-16-item short-form (DERS-16) [66] measures 6 facets of emotion regulation. The DERS-16 asks individuals to rate the extent to which 16 statements apply to them on a 5-point Likert scale, with lower scores representing higher levels of emotion regulation. The DERS-16 demonstrates adequate internal consistency (Cronbach α=.92), test-retest reliability (*r*=0.85; P<.001), and good construct validity, as evidenced by correlations with other measures of emotion regulation [66]. The total DERS-16 score was used in this study.

#### Mindfulness

Mindfulness skills were assessed using the widely used Five-Facet Mindfulness Questionnaire (FFMQ) [67], a 39-item measure capturing 5 facets of mindfulness: observing (realizing internal and external experiences such as sensations, emotions, and thoughts), acting with awareness (focusing on one’s activities in the moment as opposed to behaving mechanically), describing (labeling internal experiences with words), nonjudging of inner experience (taking a nonevaluative stance toward thoughts and feelings), and nonreactivity to inner experience (allowing the free flow of thoughts and emotions without getting caught up in by them or without rejecting them). Individuals rate each item using a 5-point Likert scale to indicate how true they believe the statement describes them; higher scores represent higher levels of mindfulness. Internal consistency of the FFMQ is shown to be good (Cronbach α between .67 and .93), and construct validity has been evidenced by correlations with meditation experience [67]. Total and subscale scores were used in this study.

#### Feasibility and Acceptability

Feasibility was assessed using study attrition rates, websites, and smartphone use data. Acceptability was explored via a satisfaction and feedback questionnaire administered to the participants upon completion of the program. The questionnaire consisted of a mixture of scaling and open-ended questions, and feedback was sought regarding content, usefulness, overall impressions, negative effects, user-friendliness, and tolerance. Detailed information about user experience of the app was collected via optional qualitative feedback phone interviews.

### Analytical Approach

Feasibility and acceptability were characterized using descriptive statistics derived from website and app use data. Evaluation of acceptability also considered descriptive statistics of participants’ responses to the web-based satisfaction and feedback questionnaire and qualitative analysis (deductive coding based on research aims) of the feedback interview. To explicate TRIBE’s central innovation of integrated use across app and web technologies, we used a behavioral archetype approach to describe the salient patterns of intervention use across the sample [47]. Behavioral archetypes were informed by objective log data on web and app activities and self-reports of use from interview data, primarily exploring the contextual factors and motivations for behavior. We expected that each participant would use the intervention elements in different ways, for example some using only the check-ins, others using the app and website quite separately, and so on, and the behavioral archetype approach was used to understand and characterize these differences in motivations and behaviors.

Finally, paired samples 2-tailed *t* tests were used to test for differences between baseline and postintervention clinical outcomes (symptoms, recovery-oriented outcomes, or process outcomes), where distributions were nonnormal, and the nonparametric alternative (Wilcoxon signed-rank test) was undertaken. Effect size was measured using Cohen *d* (parametric) or *r (*nonparametric).

## Results

### Overview

After describing the sample, 3 major types of findings are presented as follows. First, usability and feasibility feedback are presented for app and website use separately. Within each domain, we present self-reported quantitative findings as well as relevant qualitative findings including illustrative quotes. Information about any technical difficulties encountered by participants was also presented by domain. Second, integrated use of the app and website (recognizing individual differences in use) was characterized via the behavioral archetype approach. Data on overall engagement with and safety of the integrated intervention are also presented in this section. Finally, the pre- and postfindings related to the provisional efficacy of TRIBE are presented.

### Participants

A total of 56 participants registered for TRIBE and completed informed consent procedures. Of these, 33 (59%) participants responded to the initial email from assessors and were screened for inclusion in the study. Of these, 5 (15%) were excluded on the basis of an MINI diagnosis of BD-I, 1 (3%) participant withdrew partway through the study for reasons unrelated to the research, 1 (3%) participant was lost to follow-up, and 1 (3%) participant was excluded from analyses because of using an incorrect version of the smartphone app. The final sample comprised 25 (76%) participants with BD-II (19/25, 76% female participants), mean age 42.3 (SD 11.5; range 24-59) years. Participant characteristics are reported in Table 2.

**Table 2:** Participant characteristics (N=25).

Characteristics		Participants, n (%)
**Gender**		
	Female	19 (76)
	Male	6 (24)
**Country of residence**		
	Australia	6 (24)
	United States	6 (24)
	Canada	5 (20)
	United Kingdom	6 (24)
	Other	2 (8)
**Marital status**		
	Single	12 (48)
	Married	8 (32)
	Divorced	4 (16)
	Widowed	1 (4)
**Main occupation**		
	Full-time employment	7 (28)
	Part-time employment	3 (12)
	Casual employment	1 (4)
	Unemployed	3 (12)
	Full-time student	3 (12)
	Part-time student	2 (8)
	Pension	6 (24)
**Highest education level**		
	Year 11 or 12 or A levels	3 (12)
	Diploma	2 (8)
	Associate degree	2 (8)
	Bachelor’s degree	9 (36)
	Postgraduate diploma or graduate certificate	3 (12)
	Master’s degree	5 (20)
	Doctorate (PhD)	1 (4)

### Website Use and Feedback

The vast majority (23/25, 92%) of participants used the website during the 3-week intervention. The average number of sessions on the website was 9.3 (SD 8.0; range 0-32). Of these sessions,

- 63% were *Learning Only* sessions, where participants visited the website independent of the app to work through the learning materials;
- 27% were *Coping Only* sessions, where participants visited the website via the app and only accessed the coping suggestion during the session; and
- 10% were *Coping and Learning* sessions, where participants visited the website via the app, accessed the coping suggestion, and accessed learning materials.

Participants used the website for an average of 6.6 (SD 4.9; range 0-15) days and completed an average of 47% of the module content (SD 37.0; range 0-100). Participants reported that barriers to website use included time commitment required for learning, previous familiarity with content, and a current lack of symptoms. However, most participants (19/25, 76%) reported that the amount of content on the website was “about right for me” and (18/25, 72%) reported that it helped them develop new skills for managing their BD.

### App Use and Feedback

All 25 participants successfully downloaded the MoodPrism app on their phones and completed at least one check-in on the app. A maximum of 42 notifications to complete check-ins were sent to each participant (2 notifications each day for a total of 21 days); however, some participants did not receive all notifications because of technical difficulties. An average of 18 check-ins (SD 10.5; range 3-42) were completed, giving a check-in response rate of 42.9%. The average number of days participants completed check-ins across the study period was 12.4 (SD 5.6; range 3-21). Time to complete check-in items ranged from 1 to 3 minutes.

Notification-prompted mood check-ins were found to be useful by most participants (18/25, 72%), with regular mood and context ratings being reported as an “instant reminder to think about your mood” (male, aged 54 years), “[keeping] you in tune with how you are feeling” (female, aged 56 years), and being an opportunity to “just be a bit more mindful of what I was doing or be more mindful about my thoughts and feelings” (male, aged 47 years). Most participants (20/25, 80%) indicated that 2 mood check-ins per day was “about right” (with 4/25, 16% indicating they wanted more) and liked the random delivery of prompts (20/25, 80%). In terms of the mood check-in questions themselves, 84% (21/25) indicated the number of questions was “about right,” 68% (17/25) reported the questions were *completely* or *mostly* relevant, and around one-third (12/25, 48%) of participants indicated they would have liked the option to set their own check-in questions. The majority (18/25, 72%) found the mood descriptions provided by the app helpful. In addition to technical difficulties, the main barrier to completing mood check-ins when notified was because of being busy at the time (17/25, 68%). However, some participants noted that the notification itself provided an opportunity to practice mindfulness even if they were not in a position to complete the check-in at the time the notification arrived. All participants (100%) indicated that they would have liked the option to use the app outside of the prompted times.

A total of 18 out of 25 (72%) participants clicked on at least one of the links provided at the end of the check-in process to access tailored coping suggestions on the website. However, technical difficulties prevented one of these participants from accessing the website. Across the whole sample, an average of 5 links were clicked in the app across the study period (SD 7.1; range 0-24), giving a click-through from a check-in rate of 27.8%. However, in this prototype version of TRIBE, accessing the coping suggestions on the website via this process required a step of logging into the website through the app; the rate of completed click-throughs from app to website was 87.2%. Participants indicated that barriers to clicking through to the website to receive the coping suggestion included being unaware of the process of accessing the links (required clicking on the calendar presented in MoodPrism after completion of check-in), having to log into the website as part of the click-through process, being busy or not in the right environment to access or complete the suggestion, and illness-related factors (eg, did not want to be potentially triggered or felt unneeded).

Of the 2 types of coping suggestions presented after completion of a check-in, 70.6% of click-throughs were for *Try it now* suggestions and 29.4% for *Learn More* suggestions. Of the total number of website sessions that resulted from click-throughs, 26.4% incorporated additional website learning after accessing coping suggestions. Of the 17 participants who completed click-throughs to receive coping suggestions from the website, 10 (59%) reported that they followed the coping suggestion provided at least half of the time. Approximately 84% (21/25) of participants indicated that coping suggestions were useful, with participants reporting coping tips to help them bring mindfulness skills into their everyday life as well as being “helpful in helping me regain control when my anxiety levels were extremely high*.”* (female, aged 27 years) and “very on-point for the mood or feeling I was experiencing at the time.” (female, aged 47 years). However, only 44% (11/25) thought that the suggestions helped them “in the moment.”

A number of app-specific technical issues were observed during the study, with 76% (19/25) of the participants reporting technical difficulties during the study period and 28% (7/25) participants reporting some type of technical issue for >70% of the study period. The technical issues observed included device-related issues (eg, notification blocking setting on Android phones), app-related issues (eg, unavailability of check-in surveys when notifications were sent), and user-related issues (eg, lack of understanding of how to access coping prompts in apps) leading to potential reductions in the completion of check-ins and click-throughs. Participants were contacted regularly throughout the study to identify technical difficulties and assist in troubleshooting issues that were detected by the research staff during the study period.

**Figure 3:**
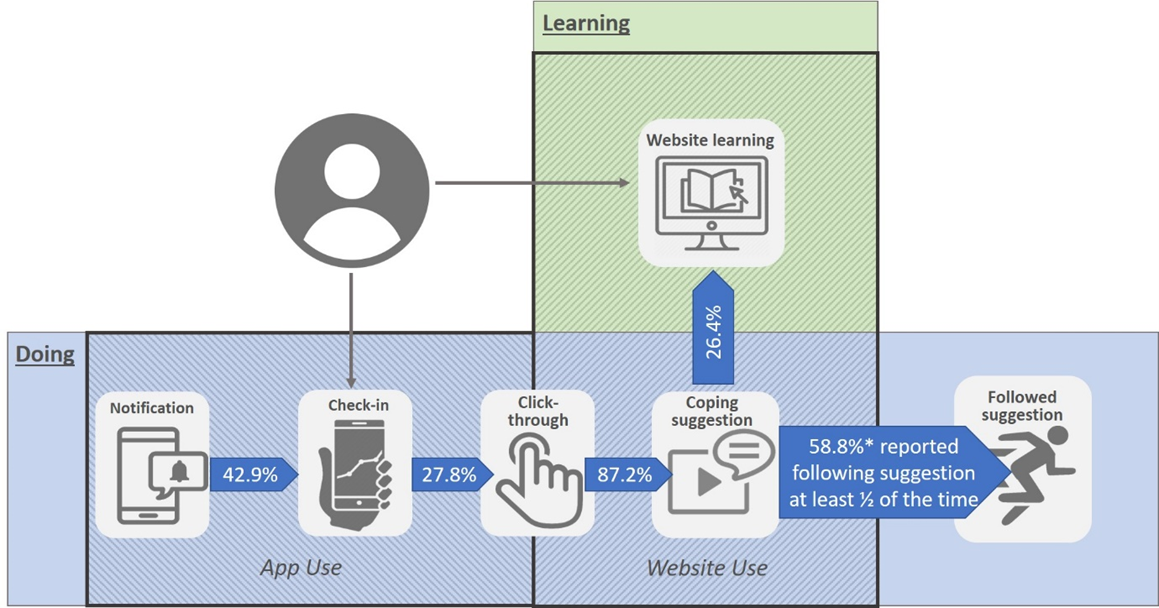
Summary of observed frequency of steps in ideal user flow journey.

A summary of the integrated use of TRIBE elements according to the ideal user interactive path model is presented in Figure 3.

### Behavioral Archetypes

**Figure 4:**
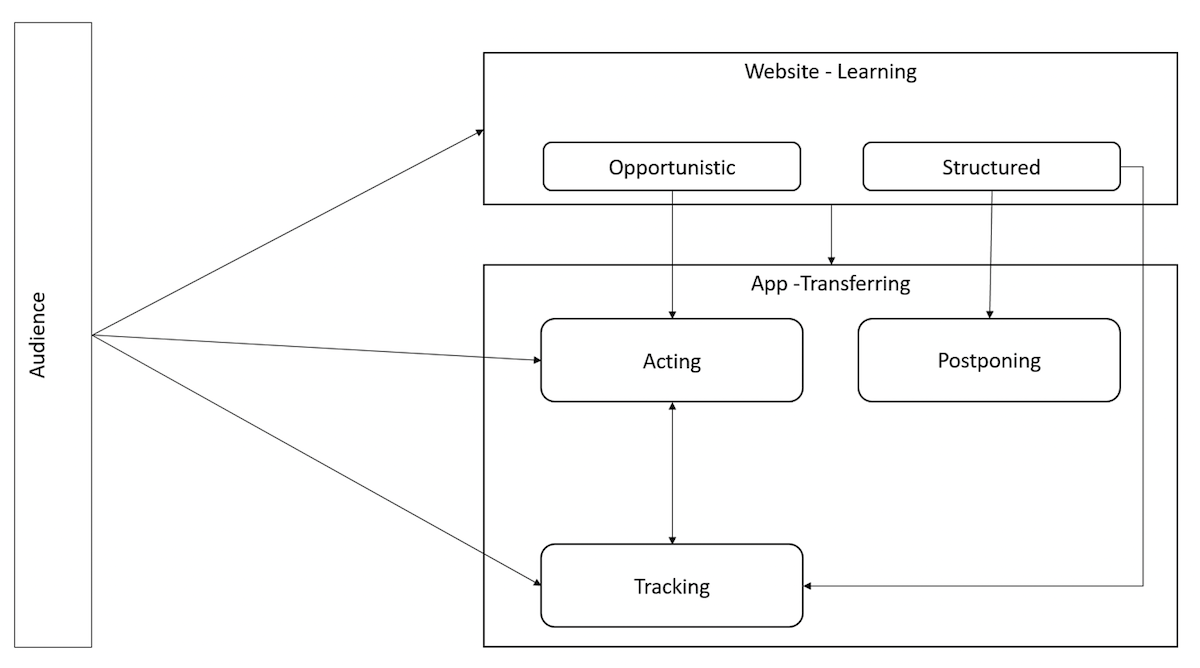
Illustrative use pathways.

Individual differences in engagement patterns were classified into 5 provisional archetypes: *structured learning*, *opportunistic learning*, *acting now*, *postponing coping*, and *tracking*. These archetypes are shown in Figure 4 and defined in Table 3. Illustrative quotes from participant feedback are provided to enrich the behavioral archetype descriptions.

**Table 3:** Identified behavioral archetypes.

Behavioral archetype	Description	Illustrative quotes
Structured learning	Participants who engaged in structured learning chose to access TRIBE^a^ website content consistently at the same times of the day and followed the course in a linear manner. Structured learning often occurred when participants were routinely constrained by time demands or were motivated to engage in the TRIBE intervention at the same time of the day to fit it in to their lives. Engaging in frequent structured learning across the study (eg, doing small chunks of content daily or every couple of days) was also linked to postponing. A subset of structured learners “top and tailed” their learning. These users completed the TRIBE website learning in single large sessions at the start and end of the intervention period. These users also engaged in the content in a linear way. Learning by top and tailing was linked to acting.	“I would tend to go on the website and do one module all at once and then come back a few days later and do the next one.” “I had quite a bad sort of manic phase where I was having kind of hallucinations and stuff like that, so I needed to not do it. So, I just didn’t do it for a while and then I filled in stuff on the website quite late on.” “I went on the website like every single day... It was usually when it’s decided I was sitting down. Okay, well let’s check my phone now. So it was a convenient time for me...”
Opportunistic learning	Participants who engaged in opportunistic learning chose to access the TRIBE website in an ad hoc manner. While completing the course in a linear way, participants did their learning components in smaller chunks across the day, when it suited them. This style of learning may have been prompted by a coping suggestion—where after viewing coping content, further opportunistic learning took place on the website by returning to the course content. Opportunistic learning had a strong link with acting.	“So you would check-in and then you would do whatever mindfulness thing it told you to do, and then you would be right there and you could learn...You could learn the stuff then, right then and there, rather than being like, ‘Oh, check-in. Okay.’ Three hours later, let’s learn about how you can manage these things and let’s learn about mindfulness. But rather, it was right then and there.”
Acting now	Acting encompassed the behavior of participants who completed both the mood check-ins and the coping prompts when they were provided. These participants were often motivated to “do something now” to alter how they were feeling. Participants who engaged in acting now sometimes transitioned into tracking behavior only.	“I found just the procedure of stopping and asking, ‘Okay what kind of mood am I in? How am I feeling?’ It really increased my awareness, made me stop and think okay what is going on and then I liked the fact that it encouraged me then to move on and try mindfulness practice...I would just be a bit more mindful of what I was doing or be more mindful about my thoughts and feelings.”
Postponing action	Postponing encompassed the behavior of participants who completed both the mood check-ins and the coping prompts, but at a later time than when it was provided by push notification. Postponing was often motivated by time or environmental context demands. Participants also postponed engagement with their coping prompt so that it aligned with their regular structured learning time. This included bookmarking behavior, where participants used previous coping prompts to access tailored content that was relevant to them at the time they chose to engage with the intervention.	“...if you’re going about your day, you know if you’re at work or if you’re on your way to somewhere you don’t necessarily have time to do a meditation right then and there, you know.” “I clicked on the ones that looked more interesting to me first, but I always went back and did the others as well eventually. So, the ones that seemed more relevant, I did them first.”
Tracking	Tracking behavior only involved use of the mood check-ins. Participants who were tracking often did so at the time it was prompted. Tracking behavior was motivated by wanting to check-in with mood but not wanting to engage with other parts of the intervention. Participants often transitioned between tracking and acting. Some participants who used structured learning only used the app intervention component for tracking.	• “Just sort of checking in with my mood did help so I felt like I didn’t need any extra help [from the website].”

^a^TRIBE: Tailored Recovery-oriented Intervention for Bipolar II Experiences.

### Feedback on the Integration of 2 Technologies

Feedback from participants is presented in Table 4. Although participants chose to use the elements offered to them in different ways, a combination of apps and websites made sense to most participants, and the majority also found the app to be a useful companion to the website. Approximately half of the participants (13/25, 52%) thought that the app helped them learn skills from the website and that the website and app were well integrated. Most participants (20/25, 80%) felt that the integrated TRIBE package helped them feel more hopeful and better manage their BD, with participants reporting that the program *“*made me think there’s more I can do about how to feel better and improve and keep your mood stable and be more resilient to things” (female, aged 27 years). In addition, most (23/25, 90%) participants would recommend it to others with BD.

**Table 4:** Satisfaction and acceptability ratings of Tailored Recovery-oriented Intervention for Bipolar II Experiences (TRIBE; N=25).

	Strongly agree, n (%)	Agree, n (%)	Neutral, n (%)	Disagree, n (%)	Strongly disagree, n (%)
The website was well integrated with the app	8 (32)	6 (24)	8 (32)	1 (4)	2 (8)
The app was a useful companion to the website	10 (40)	9 (36)	5 (20)	1 (4)	0 (0)
The combination of a website and an app made sense to me	12 (48)	8 (32)	2 (8)	3 (12)	0 (0)
The app helped me learn the skills from the website	7 (28)	6 (24)	8 (32)	4 (16)	0 (0)
TRIBE helped me manage my bipolar disorder better	6 (24)	11 (44)	6 (24)	2 (8)	0 (0)
TRIBE helped me feel more hopeful about living with bipolar disorder	8 (32)	12 (48)	3 (12)	2 (8)	0 (0)
I would recommend TRIBE to other people with bipolar disorder	13 (52)	10 (40)	2 (8)	0 (0)	0 (0)

### Engagement With the Intervention as an Integrated Whole

**Figure 5:**
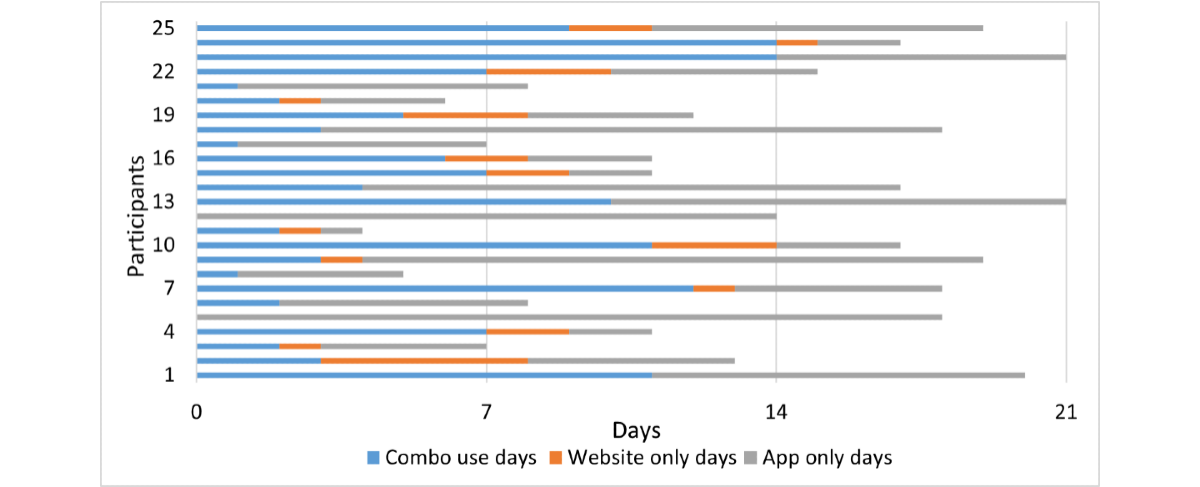
Participant engagement with Targeted Recovery-Oriented Intervention for Bipolar II Experiences.

All participants engaged in at least one element of the TRIBE process, with participants engaging on average approximately 14 out of the possible 21 days with at least one element (SD 5.3, range 4-21). Figure 5 shows the number of days that each participant interacted with website and app on the same day, website only, and app only. Approximately half (10.65/21, 51%) of the number of days participants engaged with the study were using the app only, 41% (8.63/21) with both website and app, and 8% (1.75/21) with website only. The notifications provided by the app may have also contributed to engagement with the website learning materials by acting as a reminder, even if check-ins or click-throughs were not completed, with participants reporting that “the app made me have to be more engaged on a daily basis” (female, aged 52 years).

### Safety

Although most (22/25, 88%) participants did not report any adverse events while completing TRIBE, 3 participants reported a negative impact: one indicated their mood worsened over the course of the study, attributing this in part to personal stressors and medication changes; the other found using the app *a bit stressful* because of technical difficulties; the final participant found that receiving mood check-ins when feeling depressed was confronting, as it made them think about the emotions they were experiencing (but acknowledged that this was most likely to be the time during which the coping strategies would have been useful). A participant withdrew before the follow-up assessment for reasons unrelated to the study.

### Preliminary Analysis of Efficacy

Changes in scores from baseline to after the intervention for each measure are shown in Table 5.

Mean MADRS scores decreased significantly by 2.44 points (t_24_=2.63; P=.02; Cohen *d*=0.53, 95% CI 0.5-4.36). Median YMRS score increased slightly by 1 point (*z*=−1.59; P=.11; *r*=0.22). Self-reported depression (Quick Inventory of Depressive Symptomatology-Self-Report), anxiety (DASS-Anxiety), hypomania (Altman Self-Rating Mania Scale), quality of life (QoL.BD), emotion regulation (DERS-16), and mindfulness (FFMQ) scores did not change significantly after the intervention.

**Table 5:** Pre- and postintervention scores (N=25).

Measure	Before intervention	After intervention	Difference, 95% CI	*t* test (*df*)	Z	P value	Effect size
MADRS^a^, mean (SD)	8.60 (6.86)	6.16 (5.11)	0.52 to 4.36	2.63 (24)	N/A^b^	.02	Cohen *d=*0.53
YMRS^c,d^, median (IQR)	1.00 (4.00)	2.00 (4.00)	N/A	N/A	−1.59	.11	*r=*0.22
QIDS-SR^e^, mean (SD)	7.92 (4.77)	7.44 (4.22)	−1.15 to 2.11	0.61 (24)	N/A	.55	Cohen *d=*0.12
DASS-Anx^f^, mean (SD)	4.20 (3.98)	3.96 (3.68)	−0.76 to 1.24	0.50 (24)	N/A	.63	Cohen *d*=0.10
ASRM^c,g^, median (IQR)	1.00 (4.00)	1.00 (3.50)	N/A	N/A	−.38	.70	*r*=0.05
QoL.BD^h^ scale, mean (SD)	37.64 (8.78)	39.04 (7.17)	−4.61 to 1.81	−0.90 (24)	N/A	.38	Cohen *d*=0.18
DERS-16^c,i^, median (IQR)	37.00 (26.00)	34.00 (18.50)	N/A	N/A	−1.68	.09	*r*=0.23
FFMQ^c,j^, median (IQR)	113.00 (40.00)	121.00 (37.50)	N/A	N/A	−1.81	.07	*r=*0.26

^a^MADRS: Montgomery-Asberg Depression Rating Scale.

^b^N/A: not applicable.

^c^Median, IQR, and *r* values are reported because of nonnormal distributions.

^d^YMRS: Young Mania Rating Scale.

^e^QIDS-SR: Quick Inventory of Depressive Symptomatology-Self-Report.

^f^DASS-Anx: DASS-Anxiety subscale.

^g^ASRM: Altman Self-Rating Mania Scale.

^h^QoL.BD: Quality of Life in Bipolar Disorder.

^i^DERS-16: Difficulties in Emotion Regulation Scale-16-item short-form.

^j^FFMQ: Five-Facet Mindfulness Questionnaire.

## Discussion

### Principal Findings

This study had several strengths. To our knowledge, this is the first study to test the specific utility of a technology-mediated intervention that integrates EMA/I with web-based learning modules for BD-II. The intervention content was novel, with mindfulness-based approaches specifically tailored for this population. The mixed method approach, a pretest-posttest open trial design with nested qualitative evaluation, provided rich insights into user behavior and engagement. Finally, ecological validity was maximized, with participants using their own devices in an entirely self-guided context, approximating real-world use of publicly available mental health apps.

Overall, the study results indicated that the approach is feasible and acceptable for those with BD-II. Preliminary investigation of efficacy was also encouraging. Exploration of the patterns of engagement across the 2 technologies and across time using a behavioral archetype approach generated useful insights for refinement of the platform. The pilot study was conducted *in the wild* and discussion of findings next also includes methodological insights from testing a prototype digital intervention in the full complexity of real-world application.

### Use

Adherence to daily mood check-ins was high (65.6%). Variable adherence rates have been reported in other studies adopting smartphone self-monitoring in BD, albeit over different study timeframes. For example, 55.7% adherence was reported over a 12-month period in one study [68]; in a shorter (12-week) study, adherence was 42.1% [69]; in the MONARCA II trial, adherence rates were higher (72.6%) over 9 months [70]; whereas Hidalgo-Mazzei et al [38] reported the highest rate (88%) over 3 months. Sustained user engagement is a challenge, with app use decreasing over time in other nonrandomized studies adopting ecological momentary assessment (EMA) in BD populations [38,71].

High rates of synergistic use of the app and website support the addition of EMA/I to web-based intervention in this population, with most (23/25, 92%) participants using both components on the same day and more than half (17/25, 68%) within 1 hour of each other (ie, app-prompted click-throughs to website or website triggering app use). In all, 2 (8%) participants used the app frequently but did not use the website at all during the 21-day study period. It is likely that this was because of user preference, as research suggests that individuals want a variety of functions, design elements, and self-management strategies in an app for BD, of which they can make a selection according to their personal needs [72]. Although study retention rates were high compared with other studies adopting similar app-based interventions in BD (eg, 30% at the 6-month follow-up reported by Hidalgo-Mazzei et al [73]), this may be attributable in part to the brevity of TRIBE (3 weeks).

### Acceptability

Turning to participant feedback, most participants (17/25, 68%) experienced the TRIBE intervention positively, viewing it as being helpful in managing their BD-II. Encouragingly, 80% (20/25) of participants felt more hopeful about living well with BD-II because of TRIBE, and all indicated they would recommend the program to others with BD. App features, including notification-prompted mood check-ins and coping suggestions, were rated by most (21/25, 84%) participants as useful, with some suggesting that certain features (eg, mood check-in questions) should be customizable. Flexibility and the ability to customize self-management apps for BD have been highlighted as important features by end users in other studies [74,75]. Participants indicated that they would have liked the option to use the app outside of the twice-daily prompted times, indicating a high level of engagement. These results support a user-centered approach in designing digital mental health interventions: design elements should be tailored to user needs, which may in turn promote engagement [76,77].

Qualitative data indicate that participants liked TRIBE for many reasons, including that the app notification prompts and mood check-ins lead to being more mindful, slowing their thoughts and feelings, and grounding them. Self-monitoring features within apps can increase emotional self-awareness (ie, the ability to identify and understand one’s own emotions) [78], which in turn has been shown to improve coping skills and reduce symptoms of mental illness [51,79-81]. Most participants in this study indicated that the app facilitated increased awareness of mood fluctuations, which subsequently provided space between themselves and their feelings and allowed for implementation of a coping strategy. Participants felt that this process fostered a sense of empowerment and agency in managing their moods, which is in line with previous studies that found that the perceived control of coping with prodromal symptoms and alternating mood is a key empowering element in the self-management of BD [30].

Participants appreciated that the website taught them foundational mindfulness skills and that the app then assisted them in consolidating and applying those skills to daily life. Increased engagement results in increased exposure to the intervention and potentially affects participants’ psychological health outcomes [82]. Importantly, the participants felt that the app encouraged them to engage with the website and mindful activities. Participants also described that through continued use of the app alongside the website, they began to implement mindfulness practices and coping suggestions without prompting by the app. This is reassuring given that other studies have found that continued brief weekly practice of mindfulness, following completion of a mindfulness-based intervention, is associated with improvements in depression [83].

### Efficacy

In terms of preliminary efficacy, as hypothesized, a significant reduction (with a large effect size) in clinician-rated depression (MADRS score) was observed following the intervention. Depression is the most problematic pole in BD-II, and the study results are therefore highly encouraging and warrant further testing as part of a controlled design. With regard to secondary outcomes, most shifted in expected directions with (nonsignificant) improvements in self-reported depression, hypomania, quality of life, emotion regulation, and mindfulness. Clinician-rated hypomania (YMRS score) increased slightly after the intervention; however, this effect was primarily driven by a participant who experienced increased mood elevation at follow-up. As a low-intensity 3-week intervention, it is plausible that the TRIBE intervention was not long enough to produce shifts in secondary outcomes, with limited time for skill development and consolidation. Indeed, in terms of building mindfulness skills, the amount and quality of mindfulness practice have been associated with FFMQ score changes [84]. Feedback from participants supports the testing of a longer version of TRIBE to allow adequate time for skill development:

I did not have enough relaxed time to work with the website...I wish the program lasted for a longer period of time.

Male, aged 53 years

I found the pace of the study a little too fast. Having four weeks to complete the three sections would benefit those whose life is busy.

Female, aged 46 years

The study results are interpreted with caution given the small sample size and study design (open pilot); nonetheless, there is exciting potential for the utility of EMA/I in those with BD-II, particularly given the higher mood instability for depression in this population and the associated stress, reduced quality of life, and impaired functioning [85]. Early and immediate intervention (facilitated by smartphone technology) may help temporarily avert oncoming psychopathology, empowering individuals with BD to feel more in control of their symptoms [86]. EMA/I has strong potential to support real-world generalization of skills, extending the reach and impact of web-based platforms to optimize care and specifically reducing depression in those with BD-II.

### Understanding Use Patterns via Behavioral Archetype Analysis

Behavioral archetype, or persona analysis, aims to characterize different varieties of use to support a more tailored approach to user experience. Here, the integrated web and smartphone platform enabled multiple use patterns across TRIBE’s offerings of module-based learning, EMA, and ecological momentary intervention. On the basis of automatically collected real-time use data, these patterns can be described as structured learning (logging on to website modules regularly), opportunistic learning (logging on to modules haphazardly), acting now (engaging with EMA prompts and coping suggestions at the time of the prompt), postponing action (engaging with prompts and coping suggestions when circumstances allowed), and tracking (using EMA only). These styles were not independent, with participants often shifting among use patterns (Figure 4 and Table 3). These analyses will inform the future development of TRIBE, in which a systematic co-design phase will invite end users to view involvement through these multiple archetypes and optimize the user interface to account for these different use cases. The analyses also help clarify that *intended use* of such a complex digital platform is multifaceted, and basic research may be able to identify subpopulations of BD-II who are a better fit for some forms of use over others.

### Testing a New Digital Intervention in the Real World

In addition to our aim of establishing proof of concept, this study sought to maximize its ecological validity, generating new insights about investigating prototype digital interventions. To maximize the translational significance of the findings, participants in this study were instructed to use their own device and download and install the app themselves. Furthermore, mood monitoring was entirely self-guided rather than being reviewed by a mental health professional. This design approximates how the majority of the publicly available mental health apps are used in practice, such as naturalistic and self-guided apps [52].

This *in the wild* study invited technical challenges from a user and hardware and software standpoint. Although instructions were provided on how to download, install, and use the app, user errors compromised use and user experience. Examples of this included downloading an incorrect version of the app and logging in and out of the app, resulting in front-end data loss (ie, participant being unable to view prior days of monitoring), failing to update the app (therefore experiencing *bugs* from older versions), and being unaware of the need to click on the calendar to access the mood description and associated coping suggestion. Hardware challenges included app incompatibility with certain smartphones (eg, Samsung 9s). Finally, software challenges arose in terms of Android versus iOS. For example, check-in notifications may not have been received if the *Do Not Disturb* function was set on an iPhone, whereas the Android priority system may inadvertently block notifications (requiring the user—if the issue was detected—to change settings back to *high priority* to receive notifications from the app). International time zones also present a challenge for this study. A data capture error was detected because of a mismatch between the server time (GMT+10) and the participant’s local time. This time zone mismatch also affects the delivery of push notifications. Push notifications were delivered correctly in a user’s local time, but access to the app content (mood check-in) was unlocked in the server’s time zone. As a result, participants may have received a notification to complete a mood check-in, but it was not available to them until the corresponding time was reached in the server location. Both errors required an app update to be fixed and may have disrupted the user experience for some participants. The trade-off between ecological validity and technical challenges is a key issue to consider in future smartphone-based studies. Positively, technical challenges in this study did not have a significant negative impact on end user experience. Qualitative feedback from participants indicated that they continued to draw value from the app, in particular, appreciating the fact that it reminded them to be mindful:

The very act of stopping and taking a moment to really think through every section of the questions. It really makes you practise mindfulness, and that was helpful.

Female, aged 27 years

I think sometimes I’d get a reminder and not be able to do it at the time, and I’d come back to fill in the little questions later and I wouldn’t be able to...I’d just close the app. I’d try and be mindful of how I was thinking.

Female, aged 24 years

Participants also saw value in having the app, in addition to and as a companion to the website:

It was the app that was, I feel like, cementing the importance of going to the website. So, I think, if I hadn’t had the app going off twice a day, I may have found a reason not to go and do my mindfulness.

Male, aged 54 years

### Limitations

The study’s results must be considered in the context of several limitations. As noted by other authors [85], smartphone-based studies may attract participation from individuals who are more technically oriented and motivated to complete their daily assessments. Women were overrepresented, and study participants were highly educated; the latter characteristic may affect the use of and receptivity to health technology [86]. Taken together, the study results may not be generalizable to broader BD populations. As an open pilot study, although reductions in clinician-rated depression were observed —after the intervention, the lack of an alternative intervention or control group to rule out a potential placebo effect disallows any firm conclusions to be drawn about the efficacy of the intervention. Furthermore, concomitant medication and other treatments were not assessed, limiting our ability to differentiate the efficacy of the intervention from that of routine care. The study results will inform further development of TRIBE and testing via randomized controlled trials to establish efficacy.

### Future Directions

Despite the proliferation of digital mental health interventions, efficacy studies are lacking. A multitude of smartphone apps for BD, and mental health conditions more broadly, are publicly available and have not been subjected to clinical trials [87]. Of those developed within academic settings, most have been tested in pilot designs with small populations: real-world, naturalistic studies with larger sample sizes are needed to facilitate integration of these interventions into everyday mental health care [76]. Co-design with end users in the population of interest is an essential component to promote sustained and ongoing engagement. Finally, the integration of EMA/I using integrated digital platforms (including the potential for incorporating passive collection of physiological data) is likely to extend the utility of self-management for those with BD-II and other mental health conditions characterized by chronic mood dysregulation.

### Conclusions

To our knowledge, this is the first study to test the specific utility of a technology-mediated MBI incorporating an EMA/I for BD-II. Pilot testing suggests that TRIBE is a feasible and acceptable intervention for BD-II, and preliminary efficacy results suggest that it is effective in reducing depression. TRIBE now warrants full development and evaluation as an adjunct to usual clinical care via randomized controlled trial.

## Abbreviations

BD: bipolar disorder
BD-I: bipolar I disorder
BD-II: bipolar II disorder
DASS: Depression Anxiety Stress Scale
DERS-16: Difficulties in Emotion Regulation Scale-16-item short-form
EMA: ecological momentary assessment
EMA/I: ecological momentary assessment and intervention
FFMQ: Five-Facet Mindfulness Questionnaire
MADRS: Montgomery-Asberg Depression Rating Scale
MBI: mindfulness-based intervention
MINI: Mini International Neuropsychiatric Interview
QoL.BD: Quality of Life in Bipolar Disorder
TRIBE: Tailored Recovery-oriented Intervention for Bipolar II Experiences
YMRS: Young Mania Rating Scale
